# Surface tension examination of various liquid oral, nasal, and ophthalmic dosage forms

**DOI:** 10.1186/s13065-016-0176-x

**Published:** 2016-05-09

**Authors:** Kimberly Han, Osakpolor E. Woghiren, Ronny Priefer

**Affiliations:** College of Pharmacy, Western New England University, Springfield, MA 01119 USA

## Abstract

Surface tension at the surface-to-air interface is a physico-chemical property of liquid pharmaceutical formulations that are often overlooked. To determine if a trend between surface tension and route of administration exists, a suite of oral, nasal, and ophthalmic drug formulations were analyzed. The surface tension at the surface-to-air interface of the oral formulations studied were in or above the range of the surface tension of gastric, duodenum, and jejunum fluids. The range of surface tensions for oral formulations were 36.6–64.7 dynes/cm. Nasal formulations had surface tensions below that of the normal mucosal lining fluid with a range of 30.3–44.9 dynes/cm. Ophthalmic OTC formulations had the largest range of surface tensions at the surface-to-air interface of 34.3–70.9 dynes/cm; however, all formulations indicated for treatment of dry eye had surface tensions higher than that of normal tears, while those for treatment of red eye had surface tensions below. Therefore, surface tension at the surface-to-air interface of liquid formulations is dependent on the route of administration, environment at site of introduction, and for ophthalmics, what the formulation is indicated for.

## Background

Liquid drug delivery systems undergo extensive experimental testing beyond pharmacodynamic and pharmacokinetic studies, such as pH, viscosity, density, stability, leachable studies, isotonicity, etc. A parameter that is often overlooked is surface tension at the surface-to-air interface. This is more understandable for oral or parenteral liquid dosage forms since they are quickly distributed in the physiological aqueous media of the body. However, for ophthalmic and nasal formulations, which have much greater interaction with the air, this knowledge may have a greater impact on efficiency of the delivery of the drug. Surface tension is derived from a liquid’s elastic tendency. The layer of molecules on the surface attempt to minimize their overall surface area by being attracted to molecules in the bulk liquid. It is well known that surface tension is considered a critical parameter in other areas with significant liquid–air exposures, such as spray dryer, [1] fuel injection, [2] childhood interstitial lung diseases (chILD), [3] as well as by us recently in the determination of the p*K*_*a*_ values of polymers [4] . The surface tension of ophthalmic and nasal formulations affects the rate of its evaporation, the interaction with the lacrimal film of tears or the airway mucosal lining, as well as how easily it would spread along a biological surface. To minimize irritation one would expect that liquid formulations in general would mimic the natural surface tension of the particular area of administration and thus maximize interactions. At the onset of this study we had anticipated that the surface tension at the surface-to-air interface for ophthalmic and nasal formulations would be in a very narrow range centered near that of body’s physiological environment and that oral formulations would have a much broader range of surface tensions. Herein, we report our findings on the surface tensions at the surface-to-air interface for a range of oral, nasal, and ophthalmic over-the-counter (OTC) drug substances.

## Experimental part

### Materials

Kaopectate^®^ Max (Chattem Inc), CareOne™ Regular Strength Stomach Relief (Foodhold USA, Inc), CareOne™ Loperamide HCl Oral Suspension (Foodhold USA, Inc), Children’s Delsym^®^ Cough + Cold Nitetime (Reckitt Benckiser), Chloraseptic^®^ Sore Throat (Medtech Products Inc), CareOne™ Multi-Symptom Nitetime Cold/Flu Relief (Foodhold USA, Inc), CareOne™ Non-Drowsy Daytime Cold/Flu Relief (Foodhold USA, Inc), CareOne™ Infants’ Ibuprofen Oral Suspension (Foodhold USA, Inc), Halo™ Oral Antiseptic (Oasis Consumer Healthcare, LLC), Concentrated Motrin^®^’s Infants’ Drops (McNeil Consumer Healthcare), Afrin^®^ No Drip Extra Moisturizing (MSD Consumer Care, Inc), Afrin^®^ Original Nasal Spray (MSD Consumer Care, Inc), CareOne^®^ No Drip Nasal Spray (American Sales Co), Zicam^®^ Intense Sinus Relief No Drip Liquid Nasal Gel (Matrixx Initiatives, Inc), Mucinex^®^ Sinus-Max (Reckitt Benckiser), 4 Way^®^ Nasal Decongestant Fast Acting Spray (Novartis Consumer Health, Inc), Vicks^®^ Sinex 12 Hour Decongestant Nasal Spray (Procter & Gamble), NeoSynephrine^®^ Cold & Sinus Mild Strength Spray (Bayer HealthCare LLC), CareOne^®^ Saline Nasal Spray—Nasal Moisturizing Spray (American Sales Co), Vicks^®^ Non-Drowsy Qlear Quil 12 Hour Nasal Decongestant Moisturizing (Procter & Gamble), TheraTears^®^ Lubricant Eye Drops (Advanced Vision Research, Inc), Refresh^®^ Liquigel Lubricant Eye Gel (Allergan, Inc), Refresh^®^ Optive Lubricant Eye Drops (Allergan, Inc), Systane^®^ Ultra High Performance (Alcon Laboratories, Inc), Systane^®^ Gel Drops Anytime Protection (Alcon Laboratories, Inc), Clear Eyes^®^ Contact Lens Multi-Action Relief, Systane^®^ Balance Restorative Formula (Alcon Laboratories, Inc), Visine-A^®^ Multi-Action Allergy Relief (Johnson & Johnson Healthcare Products), Clear Eyes^®^ Redness Relief (Medtech Products Inc), and CareOne™ Sterile Eye Drops (American Sales Co) were all freshly purchased from a local pharmacy, were non-expired, and used as is. Where multiple containers were need it was ensured that identical lots were employed.

### Surface tension determination

Surface tension at the surface-to-air interface was measured at room temperature in sextuplicate using a surface tensiometer (CSC-DuNOÜY, Central Scientific Co, Inc) with a 6 cm platinum tensiometer ring (Thomas Scientific). Using the correction Eq. (1), actual surface tension values were obtained.

1 $$\left( {{\text{F}}\,-\,{\text{a}}} \right)^{ 2} = {\text{ 4b}}/\left( {\pi {\text{R}}} \right)^{ 2} \, \times \,\,{\text{P}}/\left( {{\text{D}}\,-\,{\text{d}}} \right) \, + {\text{ K}}$$

where *F* = the correction factor; *R* = the radius of the ring; *r* = the radius of the wire of the ring; *P* = the apparent value or dial reading; *D* = the density of the lower phase; *d* = the density of the upper phase; *K* = 0.04534 – 1.679 *r*/*R; C* = the circumference of the ring; *a* = 0.7250; *b* = 0.0009075.

## Results and discussion

At the onset, we would like to make clear that the objective of this non-externally funded study is not to report on which drug formulation is “better” nor on which surface tension at the surface-to-air interface is “optimal” for any class of drug. To ensure this, no surface tension measurements were compared to their respective manufacturers’ reported values, if available. Furthermore, no grandiose conclusions were made regarding the efficacy of the formulations studied. We choice a du Noüy ring tensiometer for measuring the surface tension at the surface-to-air interface for this study. Briefly, a platinum ring is lowered into a solution that is being analyzed until completely submerged. Upon pulling the ring up and out of the solution, the force needed to ultimate break contact of the ring to the solution is measured. We initially began by exploring various oral formulations. We selected 10 OTC products with a range of pharmacological activity. Indications for the formulations studied include: pain/fever relief, anti-diarrheal, decongestant, antiseptic, antihistamine, indigestion, etc. CareOne™ brands were our most commonly utilized product line, not by design but rather by necessity as they had the widest range of OTC products available at the local pharmacy. We hypothesized that the surface tension at the surface-to-air interface of oral formulations would have a large range, since, upon clinical administration of the medication, the liquids would be quickly distributed throughout the significantly large volume of the GI tract. The surface tension at the surface-to-air interface at room temperature of the gastric fluids in the fasting state have been reported to be in the range of 31–45 dynes/cm (medium of 36.8 dynes/cm), [5] which is very similar to that of the duodenal fluids of ~37 dynes/cm [6, 7]. Jejunal fluids have been reported to have a slightly lower surface tension of ~30.5 dynes/cm [5]. This lowering of surface tension has been hypothesized to be due to decreased secretion of bile salts from the gall bladder compared to the duodenum (~2.5 mM compared to ~3.3 mM, respectively) [5]. There is a reported lowering of surface tension for all intestinal fluids in the fed state to 30.5 [5, 7], 31.3 [3, 5] and 30.0 dynes/cm, [8] for gastric, duodenal, and jejunal fluids respectively. In contrast, deionized water has a surface tension of 72.2 dynes/cm [9]. Since the reported surface tension of the fluids in the GI tract were determined at room temperature, the oral OTC formulations were also done at that temperature. It is known that as temperature increases, surface tension decreases. For example with deionized water there is a drop in surface tension from 71.99 to 70.41 dynes/cm as the temperature is increased from 25 to 35 °C [10]. All ten of the oral OTC formulations examined had surface tensions at the surface-to-air interface greater than the surface tension of the GI fluids at the fed state, and with one exception, above the fasting state (Table 1). Motrin^®^’s Infant Drops had the lowest surface tension of the formulations we tested, at 36.6 dynes/cm. The formulation with the highest surface tension, at 64.7 dynes/cm, was the antidiarrheal, Kaopectate^®^ Max. Non-electrolytes dissolved in an aqueous solution tend to lower the surface tension at the surface-to-air interface. Thus, it was not surprising that all formulations examined were below 72.2 dynes/cm. Compared to the other oral formulation, Kaopectate^®^ Max, CareOne™ Loperamide Hydrochloride, and CareOne™ Regular Strength Stomach Relief had the greatest surface tension values, and of those tested were the only three that contained derivatized cellulose. The most commonly employed excipient in the oral formulations were glycerin (7 liquids), propylene glycol (4 liquids), and polyethylene glycol (3 liquids). These have surface tensions of 63.4, 40.1, and 44.0 dynes/cm, [11] respectively, which partially explains the lowered surface tensions. Nonetheless, the range of surface tensions at the surface-to-air interface for the oral formulations examined was between 36.6 and 64.7 dynes/cm, or a difference of 28.1 dynes/cm.

**Table 1:** Surface tension at the surface-to-air interface for oral OTC formulations

Solution	Indication	Surface tension (dynes/cm)
Kaopectate^®^ Max	Anti-diarrheal, relives nausea and upset stomach associated with diarrhea	64.7 ± 0.1
CareOne™ Regular Strength Stomach Relief	Antihistamine for allergic reactions, motion sickness, cold, itching, nausea/vomiting, sleep aid	59.6 ± 0.6
CareOne™ Loperamide HCl Oral Suspension	Anti-diarrheal, traveler’s diarrhea	57.8 ± 0.7
Children’s Delsym^®^ Cough + Cold Nitetime	Cough, nasal congestion, symptoms of hay fever (sneezing, runny nose and itchy watery eyes)	49.0 ± 0.6
Chloraseptic^®^ Sore Throat (Phenol/Oral Anesthetic)	Upset stomach (indigestion/heartburn), anti-diarrhea, nausea, belching	44.6 ± 0.2
CareOne™ Multi-Symptom Nitetime Cold/Flu Relief	Pain/fever reducer, cough suppressant, antihistamine	41.5 ± 0.1
CareOne™ Non-Drowsy Daytime Cold/Flu Relief	Pain/fever reducer, cough suppressant, antihistamine	41.3 ± 0.2
CareOne™ Infants’ Ibuprofen Oral Suspension	Pain and fever reducer	39.0 ± 0.7
Halo™ Oral Antiseptic	Oral antiseptic	37.6 ± 0.4
Concentrated Motrin^®^’s Infants' Drops	Relieves fever and minor aches/pain due to common cold	36.6 ± 0.6
*Gastric fluid*		~*36.8 fasting* ~*30.5 fed*
*Duodenum fluid*		~*37 fasting* ~*31.3 fed*
*Jejunum fluid*		~*30.5 fasting* ~*30.0 fed*

For nasal formulations, we again selected 10 OTCs from a range of manufacturers. Not surprising, most were nasal decongestant, whether for allergies or treating symptoms of the common cold. The normal surface tension of the upper airway mucosal lining liquid has been reported to be ~56 dynes/cm [12]. The temperature of the upper airway has been reported to be slightly higher than room temperature at ~30 °C [13]. This would only produce a difference of <1 dyne/cm hence our study was conducted at room temperature. All the nasal formulations had a surface tension at the surface-to-air interface lower than the surface tension of the upper airway mucosal lining liquid (Table 2). The lower the surface tension of a solution, the less intermolecular forces present, which in turn enables it to wet a surface more readily. Similar to the oral formulations discussed above, some nasal solutions contained glycerin, derivatized cellulose, propylene glycol, and/or polyethylene glycol, all of which would lower the surface tension relative to deionized water. One ingredient that was found in all 10 nasal OTCs was benzalkonium chloride (BAC). This is typically added as a preservative, however it also a surface active agent (i.e., a cationic surfactant) [11]. The surfactant may be serving multiple roles in the formulation. Beyond a preservative, it could be increasing the solubility of the active agent, as well as allowing for the formation of micelles [11]. The latter requires that the amount of BAC added be above the critical micelle concentration (CMC). Although the quantities used in the nasal formulations are not specified, the allowable range of BAC in nasal formulations, by USP 34-NF 29 standards, is 0.002–0.02 % w/v, which could allow for micelle formation since the CMC of BAC is 0.0035 % w/v [14]. Regardless of micelle formation or not, the addition of the cationic surfactant lowers the surface tension at the surface-to-air interface. The lower surface tension of the nasal formulations allows for easier spreading over the surface, and therefore increase drug distribution and absorption.

**Table 2:** Surface tension at the surface-to-air interface for nasal OTC formulations

Solution	Indication	Surface tension (dynes/cm)
*Mucosal lining fluid*		~*56*
Afrin^®^ No Drip Extra Moisturizing	Relieves nasal congestion due to common cold, hay fever, upper respiratory allergies; swollen nasal membranes, sinus congestion/pressure	44.7 ± 0.8
Afrin^®^ Original Nasal Spray	Relieves nasal congestion due to common cold, hay fever, upper respiratory allergies; swollen nasal membranes, sinus congestion/pressure	41.6 ± 0.3
CareOne^®^ No Drip Nasal Spray	Relieves nasal congestion; sinus congestion	40.6 ± 0.4
Zicam^®^ Intense Sinus Relief No Drip Liquid Nasal Gel	Relieves nasal congestion due to common cold, hay fever, allergies, and sinusitis; sinus congestion/pressure	37.4 ± 0.6
Mucinex^®^ Sinus-Max	Relieves nasal congestion, due to common cold, hay fever, upper respiratory allergies; sinus congestion/pressure	37.2 ± 0.4
4 Way^®^ Nasal Decongestant Fast Acting Spray	Relieves nasal congestion, sinus congestion/pressure, swollen nasal membranes	36.7 ± 0.4
Vicks^®^ Sinex 12 Hour Decongestant Nasal Spray	Relieves nasal congestion due to cold, hay fever, upper respiratory allergies	33.9 ± 0.2
NeoSynephrine^®^ Cold & Sinus Mild Strength Spray	Relieves nasal congestion due to cold, allergies	33.8 ± 0.2
CareOne^®^ Saline Nasal Spray—Nasal Moisturizing Spray	Relieves dry nasal membranes	33.1 ± 0.2
Vicks^®^ Non-Drowsy Qlear Quil 12 Hour Nasal Decong. Moisturizing	Relieves nasal congestion due to cold, hay fever, other respiratory allergies; sinus congestion/pressure	30.3 ± 0.2

We had initially hypothesized that the surface tension at the surface-to-air interface for ophthalmic solutions would have the narrowest range and be very similar to that of normal tears (~43 dynes/cm) [15]. The temperature of the cornea has been reported to be only slightly higher than room temperature at <29 °C [16]. We were surprised that not only were the surface tension values of many of the ten ophthalmic OTCs substantially different than normal tears (Table 3), the range of the surface tensions was also greater than for both the nasal or oral formulations. This was initially perplexing, however, when grouped into liquids for the treatment of the symptoms of either dry eye or red eye, a pattern emerged. All the formulations that are indicated for red eye: Clear eyes^®^ Redness relief, Visine-A^®^ Multi-Action Eye Allergy Relief, and CareOne™ Sterile Eye Drops, contain a vasoconstrictor, α-adrenergic agent, naphazoline hydrochloride or tetrahydrozoline hydrochloride, which would lower the aqueous surface tension. None of the other ophthalmic drops contain a small molecule drug. As is the case for nasal sprays, a surface tension lower than that of the environment in which the solution is being introduced is beneficial to ensure greater spreading and thus absorption of the drug. In addition, the lowering of the surface tension of the ophthalmic solutions was partially due to the use of a surfactant, more accurately, BAC. Recently it has been reported that the introduction of BAC into human tears hinders the ability of native lipids to spread across the tear films and can result in the replacement of those lipid with BAC molecules [17]. It has been suggested that this phenomenon has a negative impact on the tear film stability [17].

**Table 3:** Surface tension at the surface-to-air interface for ophthalmic OTC formulations

Solution	Indication	Surface tension (dynes/cm)
TheraTears^®^ Lubricant Eye Drops	Used to moisten dry eyes and also to relieve burning sensation, irritation and discomfort caused by dry eyes	70.9 ± 0.2
Refresh^®^ Liquigel Lubricant Eye Gel	Used for moderate to severe dry eye symptoms	66.4 ± 0.2
Refresh^®^ Optive Lubricant Eye Drops	Used to relieve dry, irritated eyes. Decreases symptoms such as irritation, burning	65.6 ± 0.2
Systane^®^ Ultra High Performance	Used to relieve dry, irritated eyes. Decreases symptoms such as irritation, burning	61.0 ± 0.3
Systane^®^ Gel Drops Anytime Protection	Used to relieve dry, irritated eyes. Decreases symptoms such as irritation, burning	54.3 ± 0.6
Clear Eyes^®^ Contact Lens Multi-Action Relief	Relieves dry eyes, rewets lenses, soothes and moisturizes, removes particles from lenses	54.1 ± 0.2
Systane^®^ Balance Restorative Formula	For the temporary relief of burning and irritation due to dryness of the eye	46.5 ± 0.4
*Tears*		~*44*
Visine-A^®^ Multi-Action Allergy Relief	Temporarily relieves itchy, red eyes due to: pollen, ragweed, grass, animal hair and dander	39.5 ± 0.2
Clear Eyes^®^ Redness Relief	Relieves redness of the eye due to minor eye irritations	37.1 ± 0.2
CareOne™ Sterile Eye Drops	Relief of redness and irritation of the eye	34.3 ± 0.2

All ophthalmic OTCs tested that are indicated for dry eye have surface tensions at the surface-to-air interface above the value for tears. The three ophthalmic solutions with the greatest surface tension at the surface-to-air interface are: Thera^®^ Tears Lubricant Eye Drops, Refresh Liquigel^®^, and Refresh Optive^®^ Lubricant Eye Drops, with values of 70.9, 66.4, and 65.6 dynes/cm, respectively. These three were the only solutions tested that contained the active ingredients, sodium carboxymethylcellulose, which is used to lubricate the eye. In addition to this macromolecule salt, all three solutions contained numerous other electrolytes that contribute to higher surface tension values. Of the three products Refresh Optive^®^ Lubricant Eye Drops has the lowest surface tension. It is the only one of the three that contains glycerin, which is known to lower the surface tension values. The remaining four ophthalmic OTCs are all indicated for dry eye, and have surface tension values at the surface-to-air interface above the surface tension of tears and were in the range of 46.5–61 dynes/cm. The lubricants present in these four solutions are polyethylene glycol, propylene glycol, and/or glycerin.

## Conclusions

All OTC formulations tested had surface tensions at the surface-to-air interface less than that of deionized water. For oral formulations, there was a broad range of surface tensions, however each was at or above the surface tensions of gastric, duodenum, and jejunum fluids. For nasal formulations, all solutions had surface tension beneath the mucosal lining fluids. This allows for greater spreading of the formulation on the surface. For ophthalmic formulations, solutions indicated for treatment of red eye had surface tensions less than that of tears, presumably to allow for greater spreading. Ophthalmic solutions that were indicated for treatment of dry eye had surface tensions greater than that of tears, most likely to increase the stability of the tear film and thus allow for greater lubrication of the eye.
